# Alteration of the Gut Microbiota in Missed Abortion

**DOI:** 10.1007/s12088-023-01063-y

**Published:** 2023-02-27

**Authors:** Yi Chen, Xianqian Chen, Pingyu Chen, Xiuxia Chen, Lin Pan, Lihong Han, Tang Zhu

**Affiliations:** 1 Gynaecology Department, The First Hospital of Putian, Putian, 351100 Fujian China; 2 Key Laboratory of Translational Tumor Medicine in Fujian Province, School of Basic Medical Science, Putian University, 450 Dongzhen Road West, Putian, 351100 Fujian China; 3 Yujia Biotech., D-201, 3 Juquan Road, Guangzhou, 510700 Guangdong China

**Keywords:** Missed abortion, Gut microbiota, High-throughput sequencing, 16S rRNA

## Abstract

There is a symbiotic relationship between gut microbiota and human beings. Imbalance of the gut microbiota will cause pathological damages to humans. Although many risk factors are associated with missed abortion (MA), the pathological mechanism of it is still unclear. Here, we analyzed gut flora of the patients with MA by S16 high-throughput sequencing. The possible pathogenic mechanisms of the MA were explored. Fecal samples from 14 healthy controls and 16 MA patients were collected to do 16S rRNA gene high-throughput sequencing analysis. The abundance of the *Bacteroidetes*, *Proteobacteria*, *Actinobacteria*, *Escherichia*, *Streptococcus_ Salivarius*, and *Lactobacillus* was significantly reduced in the MA group, while, the abundance of the *Klebsiella* was significantly increased in the MA patients. The *Ruminococcaceae* and *[Eubacterium]_coprostanoligenes_group* were found only in the specimens of the MA patients. The Fabrotax function prediction analysis showed that four photosynthesis function bacteria (*cyanobateria*, *oxygenic_photoautotrophy*, *photoautotrophy*, and *phototrophy*) only existed in the MA group. In the analysis of the BugBase microbiome function prediction, the *Escherichia* of the MA group is significantly reduced compared to that of the healthy controls in the items of that *Contains_Mobile_Elements*, *Facultatively_Anaerobic*, *Forms_Biofilms*, *Potentially_Pathogenic.png*, *Gram_Nagative*, and *Stress_Tolerant_relabundance*. These alterations may affect the stability of the host's immune, neural, metabolic and other systems by interfering with the balance of the gut microbiota or by the metabolites of those bacteria, causing the MA. This study explored the possible pathogenic factors of the gut microbiota of the MA. The results provide evidence to figure out the pathogenesis of the MA.

## Introduction

Missed abortion (MA) is defined as a spontaneous death of the embryo or fetus [1, 2]. MA has a great impact on pregnant women and her families. It may cause anxiety, depression or stress in the relevant personnel. Many who experienced MA have a sad process [3]. About 80% of the MA occurs in the first three months of pregnancy [1, 4]. The risk of the MA within five to 20 weeks is 11% to 22%. The MA rate increases with the age of both parents [5, 6]. The MA rate was 11%, increased from 9% at the age of 22 to 84% at the age of 48 in Denmark [7]. The etiological cause of about half of the MA involves chromosomal abnormalities. Although scientists believe there are many risk factors, however, not all of them can be determined [8–10]. Therefore, it is still a challenge for scientists to clarify the pathological mechanism of the MA.

Gut flora is the bacteria living in the human digestive tract [11, 12]. The gut flora has a wide range of effects and perform many useful functions, such as fermenting unused energy substrates, training the immune system through metabolic end products (such as propionate and acetate, preventing the growth of harmful species), maintaining the intestinal epithelium, synthesizing vitamins (such as biotin and vitamin K) for the host, and producing hormones to guide the host to store fat, metabolize dietary and pharmaceutical compounds, control immune function and even influence behavior through gut-brain-axis. The relationship between gut flora and humans is not only symbiotic (harmless coexistence), but also reciprocal. Some human gut florae benefit the host by fermenting dietary fiber into short chain fatty acids (SCFAs) [13–15]. It has been proven that the imbalance of the gut flora will bring many diseases to humans. For example, ulcerative colitis [16], allergy, asthma and diabetes [14, 17], cirrhosis, nonalcoholic fatty liver [18], obesity [19], and even increase the risk of cancer [20]. Bacteria constitute the majority of the flora in the colon, accounting for 60% of the dry weight of feces [20]. It makes feces as an ideal source of gut flora for any test and experiment. Through nucleic acids extraction from fecal samples and 16S rRNA gene sequencing, the gut bacteriome information could be easily obtained. This method is also generally preferable to the invasive techniques such as biopsy.

About 15% of recurrent abortion is related to immune factors [21]. Autoimmune disorder is a risk factor for MA. Abnormal immune status will influence the developing fetus, resulting in abnormal embryos, leading to MA [22, 23]. On the other hand, the gut flora also has a profound impact on the host's immune system. Gut-brain-axis is a biochemical signal that occurs between the gastrointestinal tract and the central nervous system [24]. Gut flora can affect the host's neuroimmune system via the gut-brain-axis [24, 25]. Gut flora also plays a direct role in defending against pathogens, secreting cytokines to initiate an inflammatory response against infection by utilizing all available nutrients [26]. In this study, we analyzed gut flora of the patients with MA by S16 high-throughput sequence analysis. The possible pathogenic mechanisms of the MA were explored from the analysis of the differential gut flora between the patients with MA and healthy controls (EPs). The results provided a proof in gut microflora factors for the study of the pathogenic mechanism of MA.

## Materials and Methods

### Patients and Sample Collection

In this study, from June to September of 2021, 16 hospitalized early pregnant patients with the MA (average age: 29.07 ± 4.428 years old) were recruited in our hospital. 10–20 g of their fresh feces on the day of diagnosis were collected in a sterilized 50 ml covered test tube from them, kept in a − 80 °C freezer until it was submitted for gut flora detection. During the same period, the feces of 14 normal pregnant women (average age: 28.75 ± 4.235 years old) were also collected as EPs. Inclusion and diagnosis criteria of the MA were the patients whose pregnacy period were within three months and who matched the MA diagnosis of the Society of Radiologists in Ultrasound in America [27, 28]. Exclusion criteria of MA were those who had hereditary diseases, diabetes, infection, tumor, malnutrition, thyroid disease, or autoimmune diseases, who recently used antibiotics or other drugs, or who has ovarian and uterine malformations, uterine fibroids, or obesity. This study was approved by the Ethic Committee of the First Hospital of Putian (No. 2022-025). All patients and healthy controls signed the informed consents.

### Gut Flora Detection

All the gut flora were detected by Biomarker (Beijing, China). The specific primer with barcode was synthesized according to the full-length primer sequence. PCR amplification was carried out using the primers with barcode. The product was purified, quantified and homogenized to form corresponding sequencing library (Single Molecule Real Time Bell), and sequenced by PacBio Sequel RS II (Pacific Biosciences, Menlo Park, CA, USA). Cutadapt method [29] was applied to recognize the primer sequences. Trimmatic means [30] was employed to filter the CodeCharge Studio (CCS) documents. USEARCH [31] and UCHIME [32] measures were used to remove chimeras. Finally high-quality sequences were obtained for subsequent analysis.

### Operational Taxonomic Unit (OTU)/Amplification Sequence Variants (ASVs) Analysis

The sequences were clustered below the level 97% similarity using USEARCH to filter out the OTUs. The DADA2 [33] method in QIIME2 [34] was used to denoise the data after quality control. The reserved total sequences number was equal and above 0.005% Features (i.e. OTU) of the total number of sequences.

### Diversity Analysis

Alpha diversity indexes of the gut flora of the MA and the EP groups were analyzed and evaluated by QIIME2 (https://qiime2.org/) to get indexes of Chao 1 richness estimator, ACE richness estimator, Shannon Wiener diversity index, Simpson diversity index, and PD_whole_tree, respectively. The beta diversity analysis was processed with binary Jaccard, Bray Curtis and (UN) weighted UniFrac algorithms to present the species diversity matrix. R language platform was used to prepare Principal component analysis (PCA), principal coordinate analysis (PCoA) and correlation analysis between environmental factors and sample composition (RDA/CCA).

### Species Annotation and Taxonomic Analysis

The feature sequence was taxonomically annotated with Silva as the reference database, and naive Bayes classifier combined with alignment method. The species classification information corresponding to each feature and the composition of each sample colony at each level (phylum, class, order, family, genus, species) were obtained. QIIME software was applied to generate species abundance tables at different classification levels. Following that, the R language tool was used to draw the community structure map of the samples at various taxonomic levels.

### Analysis of Significance of Difference Between Groups

Significant difference analysis between groups, which can be called biomarkers analysis, at genus and species levels were performed by Metastats analysis, ANOVA and Willcox rank sum test.

### Correlation Analysis

Network graph correlation analysis was employed to perform Spearman rank correlation analysis. Screen data with correlation greater than 0.1 and *p* value less than 0.05 were used to build up a correlation network according to the abundance and change of each genus or species in each sample. The genus correlation network diagram was also drawn based on Python.

Based on the fact that the accumulation of metabolites of the microbiota may cause changes in the host microenvironment, thereby affecting the physiological and pathological activities of the host, it is necessary to study the correlation between gut flora and host metabolomic functions. On account of the analysis of network diagram, the coexistence relationship of species in environmental samples can be obtained, and the interaction and important mode information of species in the same environment can be obtained, further explaining the formation mechanism of phenotypic differences between samples.

### Function Prediction Analysis

BugBase was applied to predict the biological level coverage of functional pathways within the complex microbiome and the biologically interpretable phenotypes. Kyoto Encyclopedia of Genes and Genomes (KEGG) and FAPROTAX [35] were also employed to predict metabolic pathways and ecological relevant functions.

BugBase first normalizes OTUs by predicting 16S copy numbers, and then predicts microbial phenotypes using the provided precomputed files. First, for each sample in the biological dataset, the relative abundance of traits was estimated in the whole range of coverage thresholds (0 to 1, in increments of 0.01). Then, BugBase selects the coverage threshold with the highest variance in all samples for each feature in the data. After setting the threshold, BugBase will generate the final organism level trait prediction table, which contains the predicted trait relative abundance of each sample. The predicted phenotype types included gram positive, gram negative, biofilm forming, genetic, mobile element containing, oxygen utility, and oxidative stress tolerance.

## Results

### OTU Analysis of the S16 Sequences from the Gut Flora of Both the MA and the EP

After 30 samples were sequenced by S16 high-throughput sequence and identified by Barcode, 389045 circular consensus sequencing (CCS) sequences were obtained. Each sample generated at least 12725 CCS sequences, with an average of 12968 CCS sequences.

**Fig. 1:**
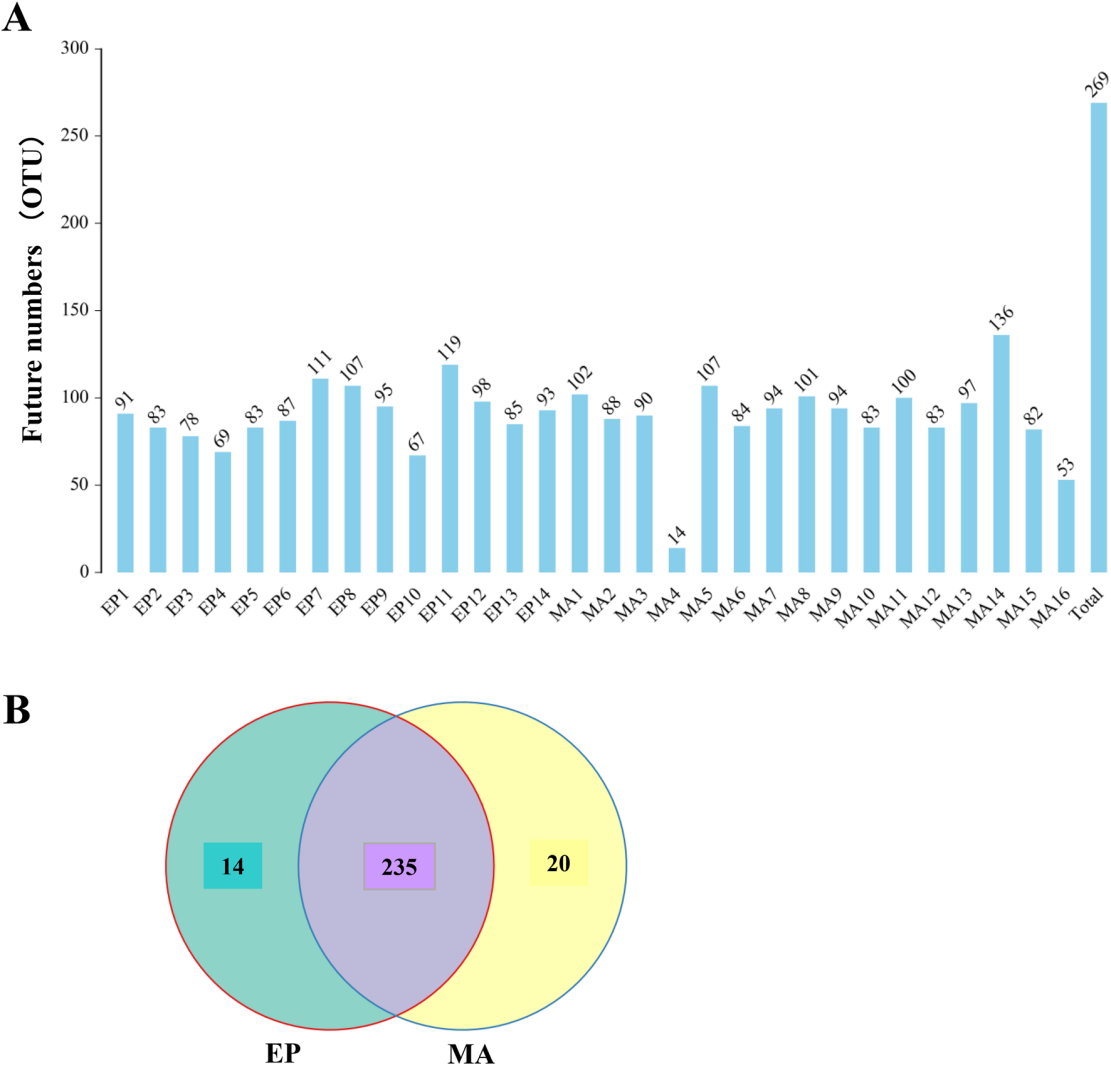
OTU analysis of the gut flora in the missed abortion (MA) samples and healthy controls (EP). **A** The OTU numbers in each MA and EP sample were indicated on top of the indicated bars. Total 269 OTU numbers found from these samples were showed in the right bar. **B** Among the 269 OTUs, 235 were shared by them (MAs and Eps). 14 were exclusively owned by Eps. 20 OTUs were specifically occupied by Mas only

Through S16 high-throughput sequence analysis, the similarity among different sequences was calculated and compared, and the fragments with 97% similarity were clustered to form different OTU. The OTU numbers found in each MA sample and EP sample were presented in Fig. 1A, respectively. In totally 269 OTUs were found among these samples. Amongst them, 235 OTUs were shared by both the MAs and EPs. 14 OTUs were exclusively owned by healthy controls and 20 OTUs were specifically shared by MAs (Fig. 1B).

### Species Annotation and Taxonomic Analysis

SILVA (https://www.arb-silva.de/) (Silva.138 version) is a comprehensive database containing rRNA gene sequences of three domain microorganisms (bacteria, archaea and eukaryotes). In order to annotate the obtained OTU data, through comparing to the SILVA database, the feature taxonomic sequence of the OTUs were annotated with the naive Bayes classifier method to obtain the species classification information corresponding to each feature. Thus, the community composition of each sample at various levels (phylum, class, order, family, genus, species) was gained. Subsequently, QIIME software was employed to generate species abundance tables at different taxonomic levels. The R language tools were used to draw community structure maps of samples at various taxonomic levels.

**Fig. 2:**
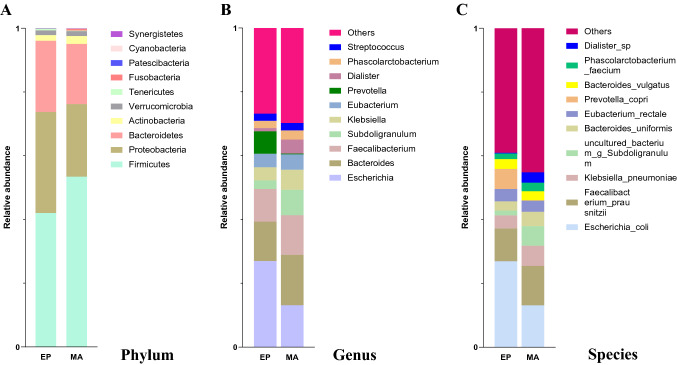
Species annotation and taxonomic analysis at genus and specie levels. The distribution of bacterial abundance at the top ten genera (Fig. 1A) and species (Fig. 1B) were presented as indicated colors in the missed abortion (MA) group and health controls (EP)

At phylum level, comparing to the EP group, the *Firmicutes* were comparatively enriched in the MA group, whereas the *Proteobacteria* and *Bacteroidetes* were relative enriched in the EP group (Fig. 2A). Based on the suggestion of Turnbaugh et al. [36] that the elevation of the *Firmicutes/Bacteroidetes* ratio is applied as a sign of pathological conditions. In our data, the ratio of the MA group was 2.83 (0.534198/0.188764) which is obviously higher than 1.883 (0.420116/0.223096) of the EP group, demonstrated that there was a pathological alteration in the MA patients.

Since the bacterial taxonomic at genus and species levels are the most abundant, in order to facilitate coherent analysis, we mainly focused on analyzing the alterations of the bacteria at the level of genus and species. In the distribution of bacterial abundance at the level of the top ten genera (Fig. 2B), compared with the EP, the relative abundance of *Escherichia*, *Subdoligranulum*, *Prevotella*, and *Dialister* in the MA group were remarkably decreased, while, the abundances of *Bacteroides* and *Klebsiella* were significantly increased in the MA group.

In the top ten distributions of bacterial abundance at specie level (Fig. 2C), compared with the EP group, the abundances of *Prevotella_copri* and *Escherichia_coli* were significantly lower, however, the abundances of *Klebsiella_pneumoniae*, *uncultured_bacterium_g_Subdoligranulum*, *Bacteroides_uniformis*, and *Dialister_sp* were obviously greater in the MA group.

### Biological Diversity Analysis

Alpha-diversity indexes analysis of Shannon, Simpson, ACE, Chao1, and PD_ehole_tree did not showed any significant differences between the MA and EP groups (*p* > 0.05) (Table 1).

**Table 1:** Alpha-index analysis between the missed abortion (MA) and health control (EP) groups (u: index; n = mean ± SE)

Indexes	EP	MA	*p* value
Shannon	3.639 ± 0.256	3.816 ± 0.218	0.6008
Simpson	0.799 ± 0.0467	0.8429 ± 0.0293	0.4203
ACE	110.6143 ± 3.6937	104.1973 ± 6.839	0.4348
Chaol	106.4329 ± 3.3567	103.7741 ± 7.878	0.7699
PD whole tree	7.9487 ± 0.2365	7.614 ± 0.4446	0.5289

Beta-diversity indexes analysis of Principal Component Analysis, Principal coordinates analysis, Non-MetricMulti-Dimensional Scaling, Unweighted Pair-group Method with Arithmetic Mean, combining UPGMA clustering tree with histogram, Sample thermographic analysis, and PERMANOVA/Anosim analysis did not showed any obvious difference between the groups as well (*p* > 0.05)(data did not show).

### Analysis of Significance of Difference Between Groups

The ANOVA and Wilcox rank sum tests revealed that the abundance of the *Synechococcus* by ANOVA analysis at genus level and the *Synechococcus_ruvescers* by Wilcox rank sum test at species level are only found in the MA group. However, the abundance of the *Acinetobacter* by Wilcox rank sum test at genus level and the *Prevotella_copri* by ANOVA analysis, the *Acinetobacter_lwoffii* and the *Synechococcus_salivarius* by Wilcox rank sum test at species level were significantly lower in the MA group compared to those in the EP group (*p* < 0.05) (Table 2).

**Table 2:** Analysis of significance of difference between groups at genus and species levels (u: relative abundance; n = mean ± SE)

Analysis	Taxonomy	Bacteria	CTL	MA	*p* value
ANOVA	Genus	Synechococcus	0 ± 0	0.000544 ± 0.000964	0.044321
Wilcox rank sum	Genus	Acinetobacter	0.001725 ± 0.004180	0.000272 ± 0.000282	0.019915
ANOVA	Species	Prevotellacopri	0.074121 ± 0.133686	0.001147 ± 0.003524	0.037164
Wilcox rank sum	Species	Acinetobacterlwo ffii	0.001525 ± 0.003893	0.000148 ± 0.000311	0.033998
Wilcox rank sum	Species	Streptococcussalivarius	0.013012 ± 0.021539	0.002925 ± 0.005856	0.019915
Wilcox rank sum	Species	Synechococcusrubesce ns	0 ± 0	0.000544 ± 0.000964	0.00882

The Metastats analysis at genus level indicated that the *Brochothrix*, the *Chryseobacterium*, the *Dielma*, and the *Mitsuokella* only existed in the EP group. The *Ruminococcaceae_UCG_008*, the *Alloprevotella*, the *uncultured_bacterium_o_Bacteroidales*, and the *Anaerotignum* were only found in the MA group. The abundances of the*[Eubacterium] _coprostanoligenes_group*, the *Brevundimonas*, the *Prevotella*, the *[Eubacterium] _ruminantium_group*, and the *Lactobacillus* were clearly lower in the MA group (*p* < 0.05). However, the abundances of the *Gemella* and the *Lachnospiraceae_ND3007_group* were significant higher in the MA group (*p* < 0.05) (Table 3).

**Table 3:** Metastats analysis of abundance difference between groups at genus level (u: relative abundance; n = mean ± SE)

Bacteria	CTL	MA	p value	q value
Ruminococcaceae_UCG-008	0 ± 0	0.000104 ± 0.000104	0.000113	0.012
Alloprevotella	0 ± 0	0.000103 ± 0.000103	0.00021	0.012
Uncultured_bacterium_o_Bacteroidales	0 ± 0	0.000096 ± 0.00001	0.000393	0.012
Anaerotignum	0 ± 0	0.00022 ± 0.00022	0.000999	0.012
Brochothrix	0.00025 ± 0.000241	0 ± 0	0.000999	0.012
Chryseobacterium	0.000629 ± 0.000629	0 ± 0	0.000999	0.012
Dielma	0.000191 ± 0.000191	0 ± 0	0.000999	0.012
Mitsuokella	0.00549 ± 0.000549	0 ± 0	0.000999	0.012
Synechococcus	0 ± 0	0.000544 ± 0.000241	0.000999	0.012
Uncultured_bacterium_c_Bacteroidia	0 ± 0	0.000194 ± 0.000194	0.000999	0.012
[Eubacterium]_coprostanoligenes_group	0.0157 ± 0.00721	0.00213 ± 0.000787	0.017	0.171
Brevundimonas	0.000317 ± 0.000184	0.000009 ± 0.000001	0.018	0.171
Prevotella	0.0823 ± 0.0381	0.00228 ± 0.00148	0.019	0.171
Gemella	0.000007 ± 0.000007	0.000169 ± 0.000104	0.02	0.171
[Eubacterium]_ruminantium_group	0.0017 ± 0.000919	0.000036 ± 0.000028	0.023	0.18
Lactobacillus	0.000718 ± 0.000381	0.000047 ± 0.000028	0.024	0.18
Lachnospiraceae_ND3007_group	0.000031 ± 0.000023	0.000181 ± 0.000088	0.046	0.324

The Metastats analysis at species level suggested that the *Brochothrix_thermosphacta*, the *Chryseobacterium_haifense*, the *Dielma_fastidiosa*, the *Lactobacillus_iners*, the *Mitsuokella_sp*, the *Mobilibacterium_massiliense*, and the *Paraprevotella_xylaniphila* were only observed in the EP group. The *Intestinibacillus_massiliensis*, the *Fusobacterium_varium*, the *uncultured_bacterium_g_Alloprevotella*, the *uncultured_bacterium_o_Bacteroidales*, and the *Anaerotignum_lactatifermentans* only existed in the MA group. The abundances of the *Prevotella_copri*, the *uncultured_bacterium_g_[Eubacterium]_coprostanoligenes_group*, the *Brevundimonas_mediterranea*, the *uncultured_bacterium_g_[Eubacterium]_ruminantium_group*, and the *Acinetobacter_lwoffii* were notably lower in the MA group compared to those in the EP group (*p* < 0.05). The abundances of the *Eubacterium_sulci*, the *Alistipes_finegoldii*, the *Gemella_sanguinis*, the *Eubacterium_ramulus*, and the *uncultured_bacterium_g_Lachnospiraceae_ND3007_group* were specifically higher in the MA group compared to those in the EP group (*p* < 0.05) (Table 4).

**Table 4:** Metastats analysis of abundance difference between groups at species level (u: relative abundance; n = mean ± SE)

Bacteria	CTL	MA	p value	q value
Intestinibacillus_rnassiliensis	0 ± 0	0.000104 ± 0.000104	0.000113	0.0134
Fus obacteriumvarium	0 ± 0	0.000113 ± 0.000102	0.00021	0.0134
Uncultured_bacterium_g_Alloprevotella	0 ± 0	0.000103 ± 0.000103	0.00021	0.0134
Uncultured_bacterium_o_Bacteroidales	0 ± 0	0.000096 ± 0.000096	0.000393	0.0134
Anaerotignum_lactatifermentans	0 ± 0	0.00022 ± 0.00022	0.000999	0.0134
Brochothrixthermosphacta	0.00025 ± 0.000241	0 ± 0	0.000999	0.0134
Chryseobacteriumhaifense	0.000629 ± 0.000629	0 ± 0	0.000999	0.0134
Dielmafastidios a	0.000191 ± 0.000191	0 ± 0	0.000999	0.0134
Lactobacillus_iners	0.000372 ± 0.000372	0 ± 0	0.000999	0.0134
Mitsuokella_sp	0.00549 ± 0.00549	0 ± 0	0.000999	0.0134
Mobilibacterium_mas siliens e	0.000109 ± 0.000109	0 ± 0	0.000999	0.0134
P araprevotellaxylaniphila	0.00023 ± 0.00023	0 ± 0	0.000999	0.0134
Prevotella ruminicola	0 ± 0	0.000192 ± 0.000192	0.000999	0.0134
Synechococcus_rubescens	0 ± 0	0.000544 ± 0.00241	0.000999	0.0134
Uncultured_bacterium_c_Bacteroidia	0 ± 0	0.000194 ± 0.000194	0.000999	0.0134
Uncultured_bacterium_g_Dialister	0 ± 0	0.00124 ± 0.00124	0.000999	0.0134
Eubacterium_sulci	0.000015 ± 0.000015	0.000105 ± 0.000064	0.00976	0.123
Alistipes_finegoldii	0.000309 ± 0.000134	0.00255 ± 0.00115	0.016	0.18
Prevotella_copri	0.0741 ± 0.0357	0.00115 ± 0.000881	0.016	0.18
Uncultured_bacterium_g_[Eubacterium]_coprostanoligenes_group	0.0157 ± 0.00721	0.00213 ± 0.000787	0.017	0.182
Brevundimonas_mediterranea	0.000317 ± 0.000184	0.000009 ± 0.000009	0.018	0.183
Gemella_sanguinis	0.000007 ± 0.000007	0.000169 ± 0.000104	0.02	0.194
Eubacterium_ramulus	0.000097 ± 0.000036	0.00116 ± 0.000665	0.023	0.205
Uncultured_bacterium_g_[Eubacterium]_ruminantium_group	0.0017 ±	0.000036 ± 0.000028	0.023	0.205
Acinetobacter_lwoffii	0.00153 ±	0.000148 ± 0.000078	0.046	0.378
Uncultured_bacterium_g_Lachnospiraceae_ND3007_group	0.000031 ± 0.000024	0.000181 ± 0.000088	0.046	0.3789

### Correlation Network Analysis at Genus Level

According to the abundance and change of each species in each sample, Spearman rank correlation analysis was conducted and data with correlation greater than 0.1 and *p* < 0.05 were screened to build a correlation network. The coexistence relationship of the species in environmental samples can be obtained. The interaction and important pattern information of the species in the same environment can be obtained to further explain the formation mechanism of phenotypic differences between samples. The highest correlation network data of the top 50 genera are summarized in Table 5(A) and (B). Most of them have positive correlation except for *Klebsiella vs Alistipes*, *Dialister vs Phascolarctobacterium*, and *Clostridium vs [Eubacterium] _ruminantium_ group* (Table 5).

**Table 5:** The highest correlation network data of the top 50 genera (*p* < 0.05)

Source	Target	Weight	Correlation
*(A)*			
Escherichia	Lactobacillus	0.5936	Positive
Bacteroides	Phascolarctobacterium	0.5862	Positive
Bacteroides	Oscillibacter	0.5702	Positive
Bacteroides	Flavonifractor	0.5737	Positive
Faecalibacterium	Roseburia	0.558	Positive
Subdoligranulum	Coprococcus	0.6197	Positive
Eubacterium	Coprococcus	0.5528	Positive
Klebsiella	Alistipes	0.5611	Negative
Prevotella	[Eubacterium]_coprostanoligenes_group	0.5945	Positive
Prevotella	Ruminococcaceae_UCG-005	0.5572	Positive
Dialister	Phascolarctobacterium	0.6155	Negative
Streptococcus	Rothia	0.7849	Positive
Streptococcus	Granulicatella	0.7262	Positive
Ruminococcaceae_UCG-014	Coprococcus_2	0.6123	Positive
Christensenellaceae_R-7_group	Ruminococcaceae_UCG-005	0.646	Positive
Christensenellaceae_R-7_group	CAG-352	0.5965	Positive
Haemophilus	Coprococcus	0.5616	Positive
Haemophilus	Lachnospira	0.5527	Positive
Alistipes	Ruminococcaceae_UCG-005	0.554	Positive
Alistipes	Butyricimonas	0.5772	Positive
Blautia	Ruminococcaceae_UCG-013	0.5496	Positive
Blautia	Romboutsia	0.5472	Positive
Blautia	Dorea	0.5994	Positive
[Eubacterium]_coprostanoligenes_group	Paraprevotella	0.5994	Positive
Ruminococcaceae_UCG-002	Ruminococcaceae_UCG-003	0.5636	Positive
*(B)*			
Erysipelotrichaceae_UCG-003	Coprococcus	0.5615	Positive
Erysipelotrichaceae_UCG-003	Collinsella	0.5941	Positive
Ruminococcac eae_UC G- 013	Butyricicoccus	0.6009	Positive
Butyricicoccus	Clostridium	0.5576	Positive
Coprococcus	Dorea	0.5727	Positive
Lachnoclostridium	Clostridium	0.5581	Positive
Lachnoclostridium	Fusicatenibacter	0.6119	Positive
Lachnoclostridium	Terrisporobacter	0.6023	Positive
Romboutsia	Fusicatenibacter	0.5901	Positive
Dorea	Collinsella	0.5835	Positive
Clostridium	Fusicatenibacter	0.5875	Positive
Clostridium	[Eubacterium]_ruminantium_group	0.5864	Negative
Clostridium	Terrisporobacter	0.5963	Positive
Megasphaera	Desulfo vibrio	0.8301	Positive
Megasphaera	Coprococcus_2	0.546	Positive
Oscillibacter	Flavonifractor	0.5888	Positive
Fusicatenibacter	Flavonifractor	0.5525	Positive
Ruminococcac eae_UC G-005	Ruminococcac eae_UC G- 003	0.6037	Positive
Veillonella	Rothia	0.5527	Positive
Ruminiclostridium 6	CAG-352	0.6372	Positive
Ruminiclostridium 6	Sutterella	0.596	Positive
Rothia	Granulicatella	0.6639	Positive
Flavonifractor	Ruminiclostridium 9	0.6545	Positive
TM7_phylum_sp._or al_clo ne_DR034	Granulicatella	0.5576	Positive
Granulicatella	Solobacterium	0.708	Positive

**Fig. 3:**
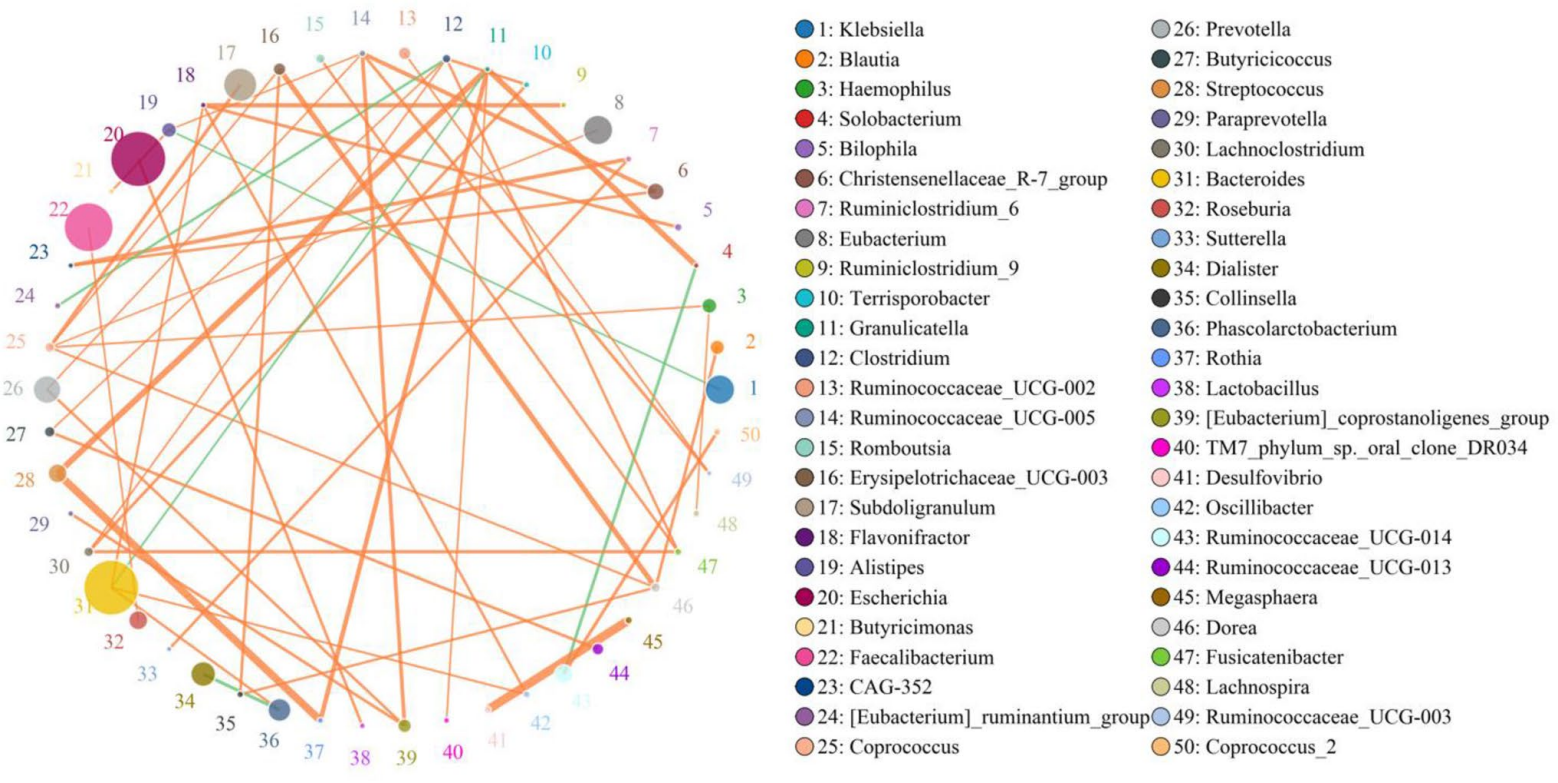
Top 50 genera correlation network based on Python analysis. The top 50 genera correlation network diagram with the highest correlation was analyzed by python. Each genus was indicated with different colors. The circle size of the genus represents the abundance of the genus. The thickness of the lines between the genera reflects the correlation strengthen

The top 50 genera correlation network diagram with the highest correlation based on Python was prepared in Fig. 3.

### Prediction and Analysis of Functional Genes at Genus Level

**Fig. 4:**
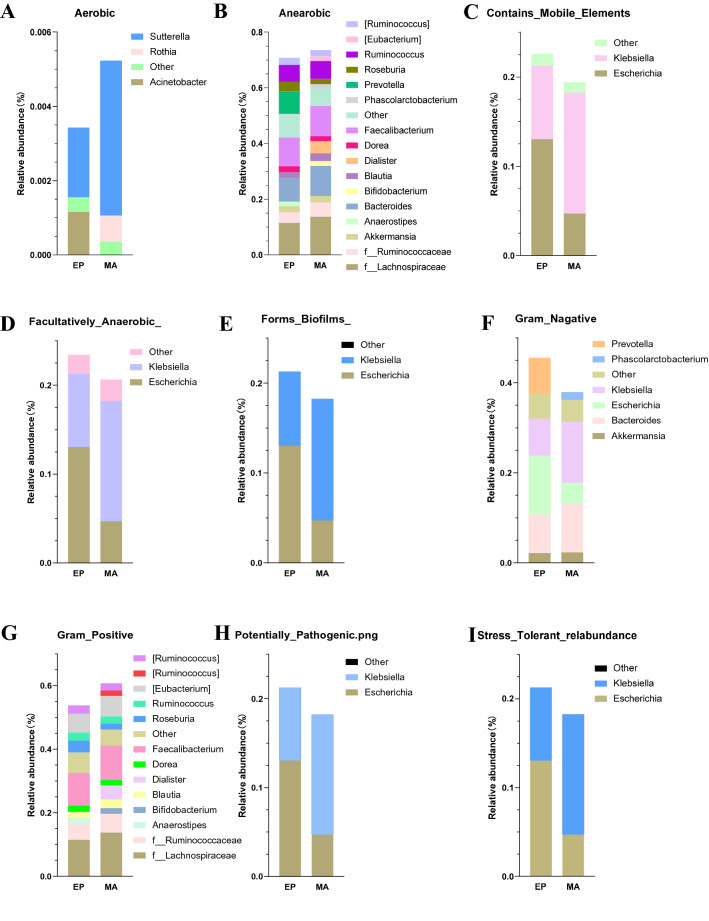
BugBase microbiome functional pathway analysis at genus level. The possible functional pathways of the gut microbiota in nine phenotypes (**A** *Aerobic*, **B** *Anearobic*, **C** *Contains_Mobile_Elements*, **D** *Facultatively_Anaerobic_*, **E** *Forms_Biofilms_*, **F** *Gram_Nagative*, **G** *Gram_Postitive*, **H** *Potentially_ Pathogenic.png*, and **I** *Stree_Tolerant_relabundance*) were analyzed by BugBase in the MA and EP groups. Different colors represent the different functions. The size of the colored bar indicates the abundance of the genus

BugBase is a method to predict the biological functional pathways in complex microbiomes and the biologically interpretable phenotype. BugBase selectively carries out automatic hypothesis testing and visualization for different traits, and generates nine phenotypes (*Aerobic*, *Anearobic*, *Contains_Mobile_Elements*, *Facultatively_Anaerobic_*, *Forms_Biofilms_*, *Gram_Nagative*, *Gram_Postitive*, *Potentially_ Pathogenic.png*, and *Stree_Tolerant_relabundance*) to describe the relative abundance of groups with characteristic traits (Fig. 4).

Further analysis of the KEGG metabolic pathway showed that the metabolic efficiency of the cell processes (i.e. cell mobility) in the MA group was significantly lower than that in the EP group (*p* < 0.05) (Table 6).

**Table 6:** Significant difference in functional gene prediction between the groups by Faprotax functional prediction (mean ± SE)

Function	EP (%)	MA (%)	p values
Cyanobacteria	0 ± 0	0.014994 ± 0.026697	0.046020773
Oxygenic_p hotoautotrop hy	0 ± 0	0.014994 ± 0.026697	0.046020773
Photoautotrophy	0 ± 0	0.014994 ± 0.026697	0.046020773
phototrophy	0 ± 0	0.014994 ± 0.026697	0.046020773

Predictive analysis of functional genes with significant difference by Faprotax suggested that the *cyanobacteria*, the *oxygenic_ photoautotrophy*, the *photoautrophy*, and the *phototrophy* were only found in MA group (Table 6).

## Discussion

Consisting with trillions of symbiotic microflorae, the gut microbiota provides the essential materials to maintain the host’s health [37, 38]. In our analyzed data, *Firmicutes*, *Proteobacteria*, *Bacteroidetes*, *Actinobacteria*, *Verrucomicrobia*, *Tenericutes*, and *Fusobacteria* were the major dominant phyla, which consists of more than 90% of the total microbial population (Fig. 2A), being consistent with the findings by Jethwani and Grover [39]. *Bacteroidetes*, *Firmicutes*, *Proteobacteria* and *Actinobacteria* are the four major phyla in human adults. The *Bacteroidetes* and the *Firmicutes* possess the highest proportion, occupying ~ 48% or ~ 51%, respectively. The *Proteobacteria* and the *Actinobacteria* are of relatively lower proportions (1%) [40]. Our results revealed that the proportion of the *Bacteroidetes*, the *Proteobacteria*, and the *Actinobacteria* in the MA group are 19%, 22%, and 2.46%, respectively, being relatively lower than those in the EP group. However, the relative ratio of the *Firmicutes* (53%) of the MA group was relatively higher than that of the EP group. (Fig. 2A) The *Firmicutes*/*Bacteroidetes* ratio is suggested to be an indicator of a pathological conditions [36]. In this study, the *Firmicutes*/*Bacteroidetes* ratio was relatively high in the MA group (2.83) compared to the EP group (1.883). The tendence of these alterations are similar to Liu's findings, although the exact ratios are not the same [41]. Firmicutes, Bacteroidetes and some anaerobic gut microorganisms can metabolize indigestible carbohydrates such as hemicellulose, cellulose, pectin, resistant starch, oligosaccharides and lignin into short chain fatty acids (SCFA), such as acetic acid, propionic acid and butyric acid [42]. The interference of the SCFA biosynthesis can cause many pathological consequences to the host [43]. Thus, the higher proportion of the *Firmicutes* (53%) may participate in the pathogenesis of the MA. The abnormal increase of the Firmicutes has been proven to be related to diabetes and obesity [44, 45]. In fact, obesity and diabetes are both the risk factors for recurrent abortion [46]. Both the observation in this experiment and Liu's study [41] clearly showed the abnormal elevation of the Firmicutes in the MA patients. Its related pathological mechanism needs further study. On the other hand, both the EP and MA groups exhibited higher rate of the *Proteobacteria* (31–22%) in our observation, which might be caused by different geography and eating habits between the Spain (< ~ 1%) and Chinese [40].

In the analysis results of the top ten microbiotas at both the genus and species levels, *Escherichia* (or *Escherichia_coli*) were significantly lower in the MA group (Fig. 2B, C). Although some *Escherichia_coli* can cause certain diseases, most of them are harmless bacteria, which can produce vitamin K2 [47] and prevent the colonization of pathogenic bacteria in the intestine, to benefit the host and to have a reciprocal relationship [48, 49]. Vitamin K2 is an essential factor for blood coagulation [50]. Disorder of coagulation is considered as one of the causes of recurrent abortion. Therefore, the abundance reduction of the *Escherichia_coli* may affect the synthesis of vitamin K2 to interfere with blood coagulation function, thus inducing the MA.

*Bacteroides* species usually constitute the most important part of the gut microbiota of mammals. Studies have shown that long-term diet is closely related to intestinal microbiome composition. Those who eat a lot of protein and animal fat are mainly *Bacteroides*, while those who eat more carbohydrates predominate with *Prevotella* [51]. In the MA cases of this study, the abundance of the *Bacteroides* (*Bacteroides_uniformis*) is higher than that of the EP group, while the abundance of the *Prevotella* (*Prevotella_copri*) is lower than that of the control group (Fig. 2B, C). This result suggests that the population of the MA group prefers a diet of large amounts of protein and animal fat. Besten et al.have shown that the *Prevotella_copri* improves insulin-sensitivity [52]. A meta-analysis indicates that insulin resistance is correlated to the susceptibility to MA, it may cause the recurrent miscarriages [53]. Thus, the decline of the *Prevotella* (*Prevotella_copri*) may participate the pathogenic process of the MA.

*Klebsiella* usually exists in human gastrointestinal tract as normal flora. However, it is also a human pathogen. *Klebsiella* can cause many diseases including pneumonia, sepsis, urinary tract infection, meningitis, peritonitis, diarrhea, and soft tissue infection. Most human *Klebsiella* infections are caused by *Klebsiella_pneumoniae* [54, 55]. The distribution of the top ten species and genera showed that the abundance of the *Klebsiella* (genus) and *Klebsiella_pneumoniae* (species) are significantly greater in the MA patients (Fig. 2B, C), suggesting that the *Klebsiella* (*Klebsiella_pneumoniae*) may play a role in the pathological mechanism of the MA.

In the difference analysis between the MA and EP groups, it also showed that the abundance of the *Synechococcus* (genus) and *Synechococcus_rubescens* (species) were significantly higher in the MA patients than those in the healthy controls at the genus and species level (Table 2). Up until now, there is no report on the pathology of the *Synechococcus* (*Synechococcus_rubescens*). Whether they play a role in the pathological mechanism of the MA remains to be seen. It is interesting to note that *Acinetobacter* and *Acinetobacter_lwoffii* are the pathogen of hospital acquired pneumonia, wound infection, bacteremia and meningitis [56]. However, they are significantly reduced in the MA patients (Table 2). *Streptococcus_ Salivarius* was found significantly lower in the MA patients compared to that in the EP group (Table 2). It belongs to a probiotic and can produce antibacterial peptide (bacteroid inhibitor), which can inhibit the growth of *Streptococcus pyogenes* [57]. It is worth noting that rheumatic heart disease is an autoimmune disease caused by *Streptococcus pyogenes*. Autoimmune disorder is also considered as a risk factor for the MA [22]. Whether the reduction of the *Streptococcus_salivarius* abundance can induce an autoimmune disorder, resulting in the MA is a subject worthy of further study.

Metastats analysis showed that the *Lactobacillus* in the MA patients were significantly lower at genus level (Table 3) and absent at species level (*Lactobacillus_iners*; Table 4). The *Lactobacillus* has been reported to regulate L-22 to maintain the host-microorganism homeostasis on the surface of intestinal mucosa and intestinal barrier function [58], prevent intestinal inflammation [59], and elevate intestinal IgA production [60]. Inflammation is considered to be one of the causes of the MA. Therefore, the reduction or deletion of the lactobacillus may also be one of the causes of the MA.

Our data shows that there was no significant difference between MA group and EP group in either alpha- or beta-diversity analysis. The results were inconsistent with those of Liu et al*.* [41]. They detected the gut flora in 41 MA patients and 19 healthy controls and found that the alpha-diversity of the MA group is significantly lower than those of the control group, indicating that the abundance and evenness of the gut flora in the MA patients are low. Whether this difference is caused by different geographical regions (Shanghai vs Putian in China), or different ages (35y vs 29y) remains to be studied.

We note that the highest correlation analysis results at the genus level showed that the *Ruminococcaceae_UCG-008* exhibited a significant positive correlation with the *coprococcus* (Table 5(A) and Fig. 3). While the *Ruminococcaceae* was not detectable in the gut flora of the normal controls (EP), however, it was found in the specimens of the MA patients (Table 3). Omura et al. [61] found a significant increase in the abundance of intestinal *coprococcus* in 35-days mice after infection with Theiler's murine encephalomyelitis virus, accompanied with increased expression of T cell receptor (TCR), IgG, IgA, various complements and major histocompatibility complex (MHC). The authors suggest that these changes are related to the damage to the nervous system caused by Theiler's murine encephalomyelitis virus infection. Therefore, the unique *Ruminococcaceae* of the MA is beneficial to the growth of *coprococcus*, which may interfere with normal fetal growth to induce MA via affecting the expression of TCR, IgG, IgA, various complements and MHC.

The highest correlation analysis results at the genus level also revealed that the *[Eubacterium]_coprostanoligenes_group* has significant positive correlation with the *Paraprevotella* (Table 5(A) and Fig. 3). Interestingly, the *[Eubacterium]_coprostanoligenes_group* was decreased in the specimens of the MA patients (Table 3). The decreased abundance of the *[Eubacterium]_coprostanoligenes_group* is not conducive to the growth of *Paraprevotella*, which may reduce the anti-infection ability of the *Paraprevotella* to pathogenic viruses. In addition, decrease of the IgA protection will also reduce the host’s immune function. All of these could be the inducing factors of the MA. Interestingly, the decrease of the *Prevotella* (Table 3) also accelerated the reduction of the *[Eubacterium]_coprostanoligenes_group* because they were positive correction (Table 5(A)), therefore, further declining the anti-infection ability in the MA patients.

It is worth noting that in the Fabrotax function prediction analysis of the difference between the two groups, only four bacteria (*cyanobateria, oxygenic_photoautotrophy, photoautotrophy,* and *phototrophy*) related to photosynthesis function existed in the MA group (Table 6). It suggests that the presence of the photosynthetic bacteria seems to be closely related to the MA. Although some *cyanobaterias* themselves contain substances of high biological value: such as polyunsaturated fatty acids, amino acids, proteins, pigments, antioxidants, vitamins and minerals [62]. However, some *cyanobaterias* can also produce cyanotoxins (including neurotoxins, cyclotoxins, endotoxins, and hepatotoxins), which are toxic to humans [63, 64]. Since the research concerning to human health and *oxygenic_photoautotrophy*, *photoautotrophy*, or *phototrophy* is almost inexitent, no comment can be made. However, this finding should be further studied to determine whether they play an important role in the pathological process of the MA.

In the microbial phenotype analysis of the BugBase microbiome function prediction, in terms of the *Contains_Mobile_Elements*, *Facultatively_Anaerobic*, *Forms_Biofilms*, *Potentially_Pathogenic.png*, *Gram_Nagative*, and *Stress_Tolerant_relabundance*, the *Escherichia* in the MA group is significantly less than that in the EP group (Fig. 4C–F, H, I). The Escherichia is considered to be related to the imbalance of female lower genital tract microbiota [65].

The limitation of this study is not able to verify the speculative pathogenic mechanism of the MA in vivo or in vitro experiments. In fact, these works need to be done in the future by multiple institutes in biochemistry, microbiology, animal model, and even clinical studies.

## Conclusion

This study reveals that there are many differences in the gut microbiota in multiple aspects between the MA and healthy controls. The proportion of the *Bacteroidetes*, *Proteobacteria*, *Actinobacteria*, *Escherichia*, *Streptococcus_ Salivarius*, and *Lactobacillus* were significantly lower in the MA group. While, the abundance of the *Klebsiella* and *Klebsiella_pneumoniae* were significantly higher in the MA patients. The *Ruminococcaceae* and *[Eubacterium]_coprostanoligenes_group* were found only in the specimens of the MA patients. The Fabrotax function prediction analysis showed that four photosynthesis function bacteria (*cyanobateria, oxygenic_photoautotrophy, photoautotrophy, and phototrophy*) only existed in the MA group. In the analysis of the BugBase microbiome function prediction, the *Escherichia* of the MA group is significantly lower compared to that of the EP group in the items of the *Contains_Mobile_Elements*, *Facultatively_Anaerobic*, *Forms_Biofilms*, *Potentially_Pathogenic.png*, *Gram_Nagative*, and *Stress_Tolerant_relabundance*. These alterations may affect the stability of the host's immune, neural, metabolic and other systems by interfering with the balance of the gut microbiota or by the metabolites of those bacteria, causing the MA.

## Data Availability

All data could be obtained by contacting to the communication author.

## Abbreviations

ASV: Amplicon sequence variant
CCS: Circular consensus sequencing
EP: Healthy controls
FAPROTAX: Functional Annotation of Prokaryotic Taxa
HPA: Hypothalamus pituitary adrenal axis
KEGG: Kyoto Encyclopedia of Genes and Genomes
*MA*: *Missed abortion*
*MH**C*: Major histocompatibility complex
*OTU*: *Operational taxonomic unit*
SCFA: Short chain fatty acids
TCR: T cell receptor

## References

[1] Wilkins LW (2012) The Johns Hopkins manual of gynecology and obstetrics, 4th edn. I. 978-1451148015

[2] Farquharson RG (2005). Updated and revised nomenclature for description of early pregnancy events. Hum Reprod.

[3] Hunter A, Tussis L, MacBeth A (2017). The presence of anxiety, depression and stress in women and their partners during pregnancies following perinatal loss: a meta-analysis. J Affect Disord.

[4] How many people are affected by or at risk for pregnancy loss or miscarriage? (2013)

[5] Ammon Avalos L, Galindo C, Li DK (2012). A systematic review to calculate background miscarriage rates using life table analysis. Birth Defects Res A Clin Mol Teratol.

[6] Kleinhaus K (2006). Paternal age and spontaneous abortion. Obstet Gynecol.

[7] Nybo Andersen AM (2000). Maternal age and fetal loss: population based register linkage study. BMJ.

[8] Vaiman D (2015). Genetic regulation of recurrent spontaneous abortion in humans. Biomed J.

[9] Ali O (2015). Term pegnancy on septate uterus: report of a case and review of the literature. Pan Afr Med J.

[10] Tersigni C (2014). Celiac disease and reproductive disorders: meta-analysis of epidemiologic associations and potential pathogenic mechanisms. Hum Reprod Update.

[11] Moszak M, Szulinska M, Bogdanski P (2020) You are what you eat-the relationship between diet, microbiota, and metabolic disorders-a review. Nutrients 12:1096 10.3390/nu12041096 PMC7230850 32326604

[12] Engel P, Moran NA (2013). The gut microbiota of insects—diversity in structure and function. FEMS Microbiol Rev.

[13] Quigley EM (2013). Gut bacteria in health and disease. Gastroenterol Hepatol (N Y).

[14] Shen S, Wong CH (2016). Bugging inflammation: role of the gut microbiota. Clin Transl Immunol.

[15] Willey JM, Sherwood L, Woolverton C (2013). Prescott's microbiology.

[16] Saxena, R., Sharma VK (2016) Medical and health genomics. In: DKs. antonarakis (ed) A metagenomic insight into the human microbiome: its implications in health and disease. Elsevier Science

[17] Gao Z (2009). Butyrate improves insulin sensitivity and increases energy expenditure in mice. Diabetes.

[18] Minemura M, Shimizu Y (2015). Gut microbiota and liver diseases. World J Gastroenterol.

[19] Ley RE (2010). Obesity and the human microbiome. Curr Opin Gastroenterol.

[20] Guarner F, Malagelada JR (2003). Gut flora in health and disease. Lancet.

[21] Hoffman B (2012) Williams gynecology. New York

[22] Carp HJ, Selmi C, Shoenfeld Y (2012). The autoimmune bases of infertility and pregnancy loss. J Autoimmun.

[23] Gleicher N, Weghofer A, Barad D (2007) Female infertility due to abnormal autoimmunity: frequently overlooked and greatly underappreciated. Part II. Expert Rev Obstetr Gynecol 2(4):465–75.

[24] Wang Y, Kasper LH (2014). The role of microbiome in central nervous system disorders. Brain Behav Immun.

[25] Dinan TG, Cryan JF (2015). The impact of gut microbiota on brain and behaviour: implications for psychiatry. Curr Opin Clin Nutr Metab Care.

[26] Yoon MY, Lee K, Yoon SS (2014). Protective role of gut commensal microbes against intestinal infections. J Microbiol.

[27] Doubilet PM (2013). Diagnostic criteria for nonviable pregnancy early in the first trimester. N Engl J Med.

[28] Xiangyin G (2020). Expert consensus on treatment of missed abortion in early pregnancy. China Pract J Gynecol Obstetr (Chinese).

[29] Martin M. Cutadapt removes adapter sequences from high-throughput sequencing reads. EMBnet J Bioinform Action 17(1)

[30] Bolger AM, Lohse M, Usadel B (2014). Trimmomatic: a flexible trimmer for Illumina sequence data. Bioinformatics.

[31] Edgar RC (2013). UPARSE: highly accurate OTU sequences from microbial amplicon reads. Nat Methods.

[32] Edgar RC (2011). UCHIME improves sensitivity and speed of chimera detection. Bioinformatics.

[33] Callahan BJ (2016). DADA2: high-resolution sample inference from Illumina amplicon data. Nat Methods.

[34] Bolyen E (2019). Reproducible, interactive, scalable and extensible microbiome data science using QIIME 2. Nat Biotechnol.

[35] Sansupa C, Wahdan SFM (2021). Can we use functional annotation of prokaryotic taxa (FAPROTAX) to assign the ecological functions of soil bacteria?. Appl Sci.

[36] Turnbaugh PJ (2006). An obesity-associated gut microbiome with increased capacity for energy harvest. Nature.

[37] Fung TC, Olson CA, Hsiao EY (2017). Interactions between the microbiota, immune and nervous systems in health and disease. Nat Neurosci.

[38] Sender R, Fuchs S, Milo R (2016). Are we really vastly outnumbered? revisiting the ratio of bacterial to host cells in humans. Cell.

[39] Jethwani P, Grover K (2019) Gut microbiota in health and diseases—a review. Int J Curr Microbiol Appl Sci (IJCMAS) 8(8):1586–1599

[40] Villaran RF (2010). Ulcerative colitis exacerbates lipopolysaccharide-induced damage to the nigral dopaminergic system: potential risk factor in Parkinson`s disease. J Neurochem.

[41] Liu Y (2021). Interactions between gut microbiota and metabolites modulate cytokine network imbalances in women with unexplained miscarriage. NPJ Biofilms Microbiomes.

[42] Louis P, Flint HJ (2017). Formation of propionate and butyrate by the human colonic microbiota. Environ Microbiol.

[43] Perry RJ (2016). Acetate mediates a microbiome-brain-beta-cell axis to promote metabolic syndrome. Nature.

[44] Ley RE (2005). Obesity alters gut microbial ecology. Proc Natl Acad Sci USA.

[45] Komaroff AL (2017). The microbiome and risk for obesity and diabetes. JAMA.

[46] Oliver A, Overton C (2014) Diagnosis and management of miscarriage. Practitioner 258: 25–8, 3 25055407

[47] Bentley R, Meganathan R (1982). Biosynthesis of vitamin K (menaquinone) in bacteria. Microbiol Rev.

[48] Hudault S, Guignot J, Servin AL (2001). Escherichia coli strains colonising the gastrointestinal tract protect germfree mice against Salmonella typhimurium infection. Gut.

[49] Reid G, Howard J, Gan BS (2001). Can bacterial interference prevent infection?. Trends Microbiol.

[50] Efsa Panel on Dietetic Products N et al (2017) Dietary reference values for vitamin K. EFSA J 15(5):e04780 10.2903/j.efsa.2017.4780 PMC7010012 32625486

[51] Wu GD (2011). Linking long-term dietary patterns with gut microbial enterotypes. Science.

[52] den Besten G (2015). Short-chain fatty acids protect against high-fat diet-induced obesity via a PPARgamma-dependent switch from lipogenesis to fat oxidation. Diabetes.

[53] Li ZL (2012). Association between recurrent miscarriages and insulin resistance: a meta analysis. Zhonghua Fu Chan Ke Za Zhi.

[54] Ristuccia PA, Cunha BA (1984). Klebsiella[J]. Inf Control Hospital Epidem.

[55] Podschun R, Ullmann U (1998) *Klebsiella* spp. as nosocomial pathogens: epidemiology, taxonomy, typing methods, and pathogenicity factors. Clin Microbiol Rev 11(4):589–603 10.1128/cmr.11.4.589 PMC88898 9767057

[56] Dent LL (2010). Multidrug resistant Acinetobacter baumannii: a descriptive study in a city hospital. BMC Infect Dis.

[57] Wescombe PA (2009). Streptococcal bacteriocins and the case for Streptococcus salivarius as model oral probiotics. Future Microbiol.

[58] Zelante T (2013). Tryptophan catabolites from microbiota engage aryl hydrocarbon receptor and balance mucosal reactivity via interleukin-22. Immunity.

[59] Lamas B (2016). CARD9 impacts colitis by altering gut microbiota metabolism of tryptophan into aryl hydrocarbon receptor ligands. Nat Med.

[60] Kaikiri H (2017). Supplemental feeding of a gut microbial metabolite of linoleic acid, 10-hydroxy-cis-12-octadecenoic acid, alleviates spontaneous atopic dermatitis and modulates intestinal microbiota in NC/nga mice. Int J Food Sci Nutr.

[61] Omura S (2020). Bioinformatics analysis of gut microbiota and CNS transcriptome in virus-induced acute myelitis and chronic inflammatory demyelination; potential association of distinct bacteria with CNS IgA upregulation. Front Immunol.

[62] Christaki E, Florou-Paneri P, Bonos E (2011). Microalgae: a novel ingredient in nutrition. Int J Food Sci Nutr.

[63] Thebault L, Lesne J, Boutin JP (1995). Cyanobacteria, their toxins and health risks. Med Trop (Mars).

[64] Weirich CA, Miller TR (2014). Freshwater harmful algal blooms: toxins and children's health. Curr Probl Pediatr Adolesc Health Care.

[65] Bennett J (2015). Mandell, Douglas, and Bennett's principles and practice of infectious diseases.

